# The risk of rabies spread in Japan: a mathematical modelling assessment

**DOI:** 10.1017/S0950268818001267

**Published:** 2018-05-21

**Authors:** H. Kadowaki, K. Hampson, K. Tojinbara, A. Yamada, K. Makita

**Affiliations:** 1 Veterinary Epidemiology Unit, Division of Health and Environmental Sciences, Department of Veterinary Medicine, School of Veterinary Medicine, Rakuno Gakuen University, 582 Bunkyodai Midorimachi, Ebetsu, 069-8501, Japan; 2 Institute of Biodiversity, Animal Health and Comparative Medicine, College of Medical, Veterinary & Life Sciences, Graham Kerr Building, University of Glasgow, Glasgow G12 8QQ, UK; 3 Tojinbara Veterinary Service, 324-4 Fukutawara, Tougane, 283-0812, Japan; 4 Laboratory of Veterinary Public Health, Graduate School of Agriculture and Life Sciences, University of Tokyo, 1-1-1 Yayoi, Tokyo, 113-8657, Japan

**Keywords:** Mathematical modelling, rabies (animal), veterinary epidemiology

## Abstract

Rabies was eliminated from Japan in 1957. In the 60 years since elimination, vaccination coverage has declined and dog ownership habits have changed. The purpose of this study was to assess the current risk of rabies spread in Japan. A spatially explicit transmission model was developed at the 1 km^2^ grid scale for Hokkaido and Ibaraki Prefectures. Parameters associated with dog movement and bite injuries were estimated using historical records from Japan, and were used with previously published epidemiological parameters. The final epidemic size, efficacy of rabies contingency plans and the influence of dog owner responses to incursions were assessed by the model. Average outbreak sizes for dog rabies were 3.1 and 4.7 dogs in Hokkaido and Ibaraki Prefectures, respectively. Average number of bite injury cases were 4.4 and 6.7 persons in Hokkaido and Ibaraki Prefectures, respectively. Discontinuation of mandatory vaccination increased outbreak sizes in these prefectures. Sensitivity analyses showed that higher chance of unintentional release of rabid dogs by their owners (from 0.5 to 0.9 probability) increased outbreak size twofolds. Our model outputs suggested that at present, incursions of rabies into Japan are very unlikely to cause large outbreaks. Critically, the reaction of dog owners to their dogs developing rabies considerably impacts the course of outbreaks. Contingency measures should therefore include sensitisation of dog owners.

## Introduction

Rabies is a fatal zoonotic disease. Every year 59 000 people are estimated to die of rabies transmitted by domestic dogs, mostly in Asia and Africa [1]. The main reservoir hosts for rabies are domestic dogs in low- and middle-income countries, but wild animals including foxes, raccoons, skunks and raccoon dogs also maintain rabies in some parts of the world [2]. Historically, measures for controlling dog rabies have included dog population management, movement restriction and vaccination [3]. Dog vaccination is the most effective control measure [4, 5]. Today, rabies mostly circulates in countries where large-scale dog vaccinations have not been undertaken, and has emerged in unvaccinated dog populations in previously rabies-free areas [6, 7].

Japan has been free from rabies since 1957 [8], following rigorous implementation of control measures under the Rabies Prevention Act, which was enacted in 1950. This act includes dog registration, capture of free-roaming dogs, mandatory dog vaccination and quarantine of animals brought into the country. In Japan, free-roaming dogs can still be found, and they are captured by local government employees. Average number of captured dogs per year in entire Japan between 2012 and 2016 was 45 193 [9]. Dog vaccinations were performed twice per year until 1985, and dog vaccination coverage remained high until 1995 [10]. The long absence of rabies from Japan has weakened public interest in rabies control measures, however, and based on reported numbers of vaccinated dogs and the estimated total dog population in Japan, including non-registered dogs, overall vaccination coverage in Japan has declined to <50% [10, 11].

There is therefore growing concern about the elevated risk of rabies spreading following the introduction of a rabid dog into Japan – though the risk of such an introduction is considered to be very low [8]. Compared with the time when rabies was endemic in Japan, many more dog owners confine their dogs inside and the number of stray dogs has substantially reduced [9, 12]. Therefore, the current risk of rabies spreading in Japan has likely changed considerably compared with the historical risk. The aims of our study were to assess, using infectious disease modelling, (1) the risk of rabies spread in Japan in the event of a rabid dog being introduced, and (2) the efficacy of current contingency plans to prevent an outbreak, which include epidemiological survey to detect suspected dogs contacted with an index and subsequent cases, emergency vaccination and capture of free-roaming dogs.

## Methods and materials

### Study site selection

**Fig. 1.**
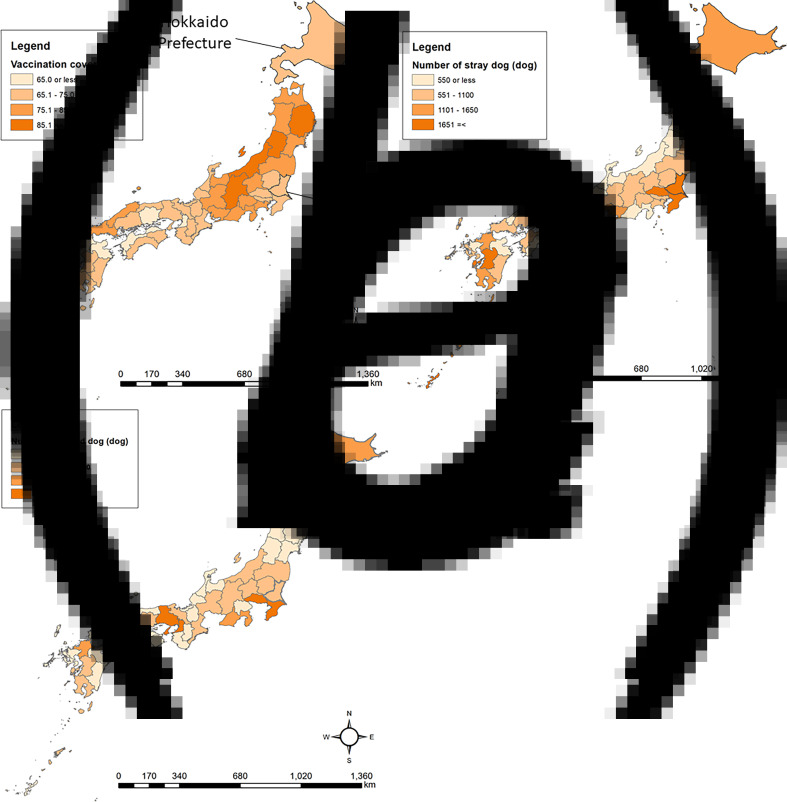
Hokkaido and Ibaraki Prefectures in Japan and potential high-risk characteristics of prefectures, specifically: (a) the number of owned dogs, (b) the number of stray dogs and (c) vaccination coverage.

Ibaraki and Hokkaido Prefectures were selected for this study (Fig. 1). Ibaraki Prefecture was chosen because vaccination coverage is low (51.8%, Table 1), and more free-roaming dogs are captured annually in the prefecture than any other in Japan [11, 12]. Hokkaido Prefecture was also chosen because of the frequency of visits to its ports by fishing vessels from Russia, where rabies is endemic [8]. In Hokkaido Prefecture, estimated vaccination coverage is higher than in Ibaraki Prefecture (56.3%, Table 1), but many free-roaming dogs are captured annually [9, 11].

**Table 1.** Characteristics of Hokkaido and Ibaraki Prefectures

Items	Hokkaido	Ibaraki
Area (km^2^)	77 282	5878
Human population	5 454 447	2 941 109
Human population density (/km^2^)	70.6	500.4
Number of stray dogs	1089	2181
Estimated total dog population	352 662	232 683
Density of dogs (dogs/km^2^)	4.6	39.6
Vaccination coverage (%)	56.3	51.8
Proportion of dogs registered (%)	80.8	79.0

### Data collection

Demographic and epidemiological data including numbers of registered dogs, vaccination coverage among registered dogs and numbers of stray dogs captured per city/town/village (shi/cho/son) administrative unit in 2013 were collected from both prefectures. Numbers of captured stray dogs may not accurately reflect total numbers of stray dogs in an area, but was used as a best-estimate proxy. Vaccination coverage was calculated from the recorded number of vaccinated dogs and the estimated total dog population [11].

This study used historical data on rabies epidemics in Osaka Prefecture between 1914 and 1915, from prefectural reports and newspapers, described elsewhere [13]. The 1914 dog population was collected from newspaper reports [13] and the human population from the 1920 census [14].

To estimate the public health impact of rabies, public libraries were visited, and newspaper and public reports published in Tokyo and the surrounding prefectures between 1893 and 1954 were surveyed for descriptions of the numbers of persons bitten by individual rabid dogs. The newspaper and public records describing number of bite injury cases by a single rabid dog in detail were extracted from these data and were used for the analysis.

### The model

We used an individual-based model to simulate the spread of rabies on a 1-by-1 km grid representing the geography of the study prefectures, as adapted from a previous study [5]. Each cell included demographic and epidemiological information, such as the human and dog population densities and vaccination coverage within the city/township administrative unit. Dog population was assumed to be static in the model because majority of owned dogs are sterilised or castrated and kept inside in Japan. Although stray dogs are less neutered (previously owned stray dogs may be neutered), contribution of turnover of stray dogs to entire dog population in Japan is limited because stray dog population is much smaller than that of owned dogs. Moreover, the period of interest for this risk assessment is first several months until the elimination after a rabies incursion.

For every simulated case, the number of secondary cases was determined by drawing from a negative binomial distribution with mean equal to the basic reproductive number, *R*_0_. Secondary cases are allocated to grid cells according to a dispersal kernel that was estimated from a study in Tanzanian [15] and historical record in Osaka, and become infectious after the serial interval, *T*_s_, elapses. Estimation of *R*_0_ and *T*_s_ (Table 2) from historical epidemics in Osaka Prefecture is described elsewhere [13]. All analyses were conducted using R, version 3.1.0 [18].

**Table 2.** Model parameters and distributions used in the default model (Ibaraki Prefecture with vaccination coverage at 0%)

Items	Parameters/distributions (unit)	Source
Reproduction number	Gamma distribution (shape = 24.6, rate = 9.87) (dog); *R*_0_ = 2.42 (90% CI 1.94–2.91)	[13]
Serial interval	Gamma (shape = 1.41, rate = 0.03) (day)	[13]
Dispersion kernel	Gamma (shape = 0.215, scale = 4.08) (km)	[16]
Mixing parameter (*α*)	0.5	Estimated
Adjusting parameter for dog population (*δ*)	0.73	Estimated
Adjusting parameter for dispersion kernel (*ε*)	1.13	Estimated
Walking time with dog per day	Beta (shape 1 = 0.01, shape 2 = 0.5) (h)	[17]
Delay of initial action	30 days	Assumption
Probability of detection in outbreak investigation	Binomial (*P* = 0.5, *n* = *N*_det_) (dog)	Assumption
The number of captured dogs	Poisson (*λ* = 10) (dog)	Assumption
The number of vaccinated dogs	Poisson (*λ* = 100) (dog)	Assumption
Probability of releasing a rabid owned dog	50%	Assumption
Probability that a rabid stray dog selects a stray dog to bite	50%	Assumption

### Initialisation

The total number of owned and stray dogs staying outside at a given time in each grid cell is denoted dogs_*i*_ and cases cannot exceed this number. To calculate dogs_*i*_, dogs were classified into: (*a*) dogs that are always kept indoors, (*b*) dogs that go outdoors only for walks, (*c*) dogs occasionally kept outdoors, (*d*) dogs always kept outdoors and (*e*) stray dogs, and the registered dogs in each administrative unit were proportionally categorised into *a*, *b*, *c* and *d* according to the published data that estimated the proportions of owned dogs in these categories [12]. Dogs were assigned within a cell according to these proportions, with the estimated number of dogs in a given administrative unit equally allocated across the cells within that unit. Dogs in category *a* were assumed to never have contact with a rabid dog and were removed from dogs_*i*_. The number of dogs outdoors in category *b* (*N*_Out*,i*_) at a given time was modelled using a binomial distribution, with the number of dogs in category *b* in cell *i N*_*b*,*i*_, and the probability of being outdoors at a given time *P*_W_, calculated from published data on daily time spent walking [15]:
1
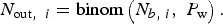


Stray dogs (*e*) in each administrative unit were allocated randomly to the cells within it. Thus, dogs_*i*_ is described as:
2
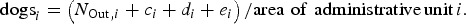

For category *c*, the published data [12] did not show how often the dogs were outside, and dogs in category *c* were dealt as same as *d* in the model.

When an owned dog was bitten, the vaccination status of the dog was stochastically assigned using the vaccination coverage of the model. Vaccinated animals were assumed not to develop rabies if bitten by a rabid dog. Stray dogs were assumed to all be unvaccinated.

### Parameterisation

Locations of secondary cases were assigned according to the distances from primary cases assuming preferential movement towards areas of higher dog population density, as previous study showed a significant positive relationship between the number of dog rabies cases and dog density [13]. Therefore, the probability of secondary cases occurring in cell *i*, Pr_*i*_ was:
3
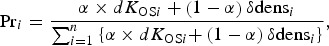

where: *dK*_OS*i*_ is the likelihood of the distance between primary and secondary cases, according to the dispersal kernel derived from the historical epidemic in Osaka [13]; dens_*i*_ is the dog population density in cell *i*; the parameter *α* characterises the influence of dog population density on secondary case locations, taking a value between 0 and 1; and *δ* is a tuning constant. *dK*_OS_ was modelled modifying a *γ* distributed dispersal kernel reported from Tanzania [17]. The shape parameter of a *γ* distribution represents numbers of events, and the scale parameter the time duration [16], or distance for a dispersal kernel. Thus *dK*_OS_ was modelled as:
4
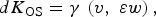

where *ε* adjusts the distance between primary and secondary cases in Tanzania [17] to the historical Osaka epidemic [13]. The distance between primary and secondary cases in Osaka Prefecture was calculated from the centroids of the administrative units where cases occurred, as exact locations were not available. Dog population data at the zone/township/village administrative unit were not available, but only the total dog population for Osaka Prefecture. A constant dog-to-human ratio was therefore assumed for all locations, based on the dog population in 1914 described in a newspaper as over a hundred thousand dogs and human population census in 1920 [14] (100 000 *vs.* 2 887 496). Dogs were allocated to each unit accordingly. The parameters *α*, *ε* and *δ* were optimised using the optim() function of R [18] to minimise the *χ*^2^ value:
5
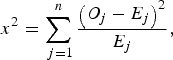

where *n* is the total number of administrative units in Osaka Prefecture in 1914 and *O*_*j*_ the number of reported rabies cases in administrative unit, *j*. Expected rabies cases, *E*_*j*_, in each administrative unit, with a particular parameter set were obtained from 1000 iterations of the simulation. Estimated parameters were validated by using the *χ*^2^ test to verify that there was no significant difference between the numbers of simulated and reported cases in each administrative unit [19].

Two other aspects relating to the behaviour of rabid dogs and their owners was modelled. In Japan, owned dogs kept indoors are confined and those kept outdoors are leashed. Therefore, if the owner of an infected dog does not release it, the risk of secondary cases is negligible. However, a rabid dog may accidentally be released by its owner, therefore unintended release was modelled. If a stray dog becomes rabid, the dog may tend to bite stray dogs, which are more accessible than owned dogs. Rabid owned dogs were modelled to bite owned and stray dogs with probabilities corresponding to the proportions of owned and stray dogs in each cell; whereas, rabid stray dogs were modelled to bite stray and owned dogs with equal probability despite a greater proportion of dogs being owned. This assumption was made because stray dogs remain in remote and non-residential areas in Japan, and they tend to live in groups of stray dogs.

### Risk assessment

To assess the current risk of dog rabies spread, vaccination coverage in the contemporary dog population was used. The probability of unintended release of a rabid dog was set as 50%, as there was no prior information on this probability. The models for Hokkaido and Ibaraki Prefectures were each run for 1000 iterations, and outbreak sizes recorded when all cases had either died or been captured. To assess the efficacy of rabies control measures, vaccination coverage was reduced to 0% to reflect discontinuation of the current mandatory vaccination scheme and compared with the status quo.

The initial response to a rabies outbreak is, by law, an immediate epidemiological investigation by the prefectural government to search for dogs contacted by the index case. This involves detection of secondary dog rabies cases, capture of stray dogs and emergency vaccination of dogs around the area of the detected case(s). Four outbreak response parameters were explored to assess the efficacy of contingency plans: (1) time until the response, (2) ability to detect rabid dogs and dogs contacted by rabid dogs, (3) speed of capturing stray dogs and (4) speed of emergency vaccination. In this assessment, only Ibaraki Prefecture was modelled, as Hokkaido Prefecture has much larger land and computation time was very long. Vaccination coverage was set to 0% because under current coverage, too few cases were generated to assess the effect of outbreak responses.

The daily number of detected rabid dogs and dogs contacted by rabid dogs was modelled using a binomial distribution, where the total number of rabid and contacted dogs in a cell is denoted as *N*_Cont_ (Table 2). Investigations were modelled as being implemented within a 5 km radius from the centroid of the cell with the detected case, on the basis of discussions with responsible prefectural government officers. Both the speed of capturing stray dogs and of emergency vaccinations were modelled as the number of dogs captured or vaccinated per day, drawn from Poisson distributions (Table 2). We assumed that vaccinated dogs became immune 14 days post-vaccination.

Different scenarios were tested to assess the efficacy of contingency plans, agreed upon as plausible scenarios through discussions with prefectural government officers (Table 3). Each scenario was simulated 1000 times and each index case was randomly generated.

**Table 3.** The parameter set of each scenario for rabies control options

Scenario	Change of parameter values
Delay of initial response	Start of initial response at day 90
Increasing capacity of detecting rabid and contacted dogs	Probability of detection: 80%
Increasing capacity of capturing stray dogs	Capturing 20 dogs per day
Increasing capacity of emergency dog vaccination	Vaccinating 200 dogs per day

To assess the risk of rabid dog bite injuries, two approaches were taken: ((1) stochastic approach) each rabid dog generated in the model was assigned either it bit at least one person or not, and the number of people to bite if the dog was assigned to bite, and ((2) ratio approach) according to the bite injury cases – rabid dog ratio (0.83 persons/rabid dog; 3805 victims *vs.* 4584 rabid dogs) reported in the past epidemic in Osaka Prefecture [13], the number of bite injury cases was calculated using the dog rabies outbreak size for each simulation. For the stochastic approach, rabid dogs were stochastically assigned to bite with the probability of 25.7% based on the published report (of 1000 rabid dogs, 743 dogs do not bite people) [20], and the number of bite injuries by a rabid dog was modelled using bootstrapping of 116 historical records from Tokyo. For each iteration scenario of rabies incursion, above-mentioned assignment of the numbers of human bite injuries for all the rabid dogs was done for 1000 times, which produced 1000 scenarios of total numbers of bite injuries by an incursion. As final epidemic size of dog rabies was simulated for 1000 iterations, in total 1 million estimates of numbers of bite injuries were generated, and injuries per outbreak summarised (mean, median and 2.5th and 97.5th percentiles), for both prefectures.

### Sensitivity analysis

The sensitivity analyses were performed for four parameters using the default Ibaraki model under current vaccination coverage: *R*_0_, vaccination coverage, the unintended release of a rabid dog by their owner and preferential contact by rabid stray dogs. The value space of the four parameters was summarised in Table 4. Smaller values than the default setting at 0.4 intervals were prepared for *R*_0_, as the values reported worldwide tend to be smaller than our default [17]. The probability that a rabid stray dog selects a stray dog to bite, 0.9% was taken from the proportion of stray dogs among all dogs in Ibaraki Prefectures, so that the selection behaviour for a stray rabid dog becomes equivalent of that of an owned rabid dog. Ibaraki Prefecture model with current vaccination coverage was used for the sensitivity analyses, and the 1000 iterations were performed for each condition.

**Table 4.** Scenario analysis results of final size and duration of the epidemic in Ibaraki Prefecture (vaccination coverage: 0%)

Scenario	Final size			Epidemic period in days (95% CI)
	Mean (95% CI)	Range	Probability of secondary cases (95% CI)	
Default setting	21.7 (1–110)	1–152	35.1% (32.2–38.2)	152.5 (1–581)
Delayed initial response	22.9 (1–119)	1–157	38.6% (35.6–41.7)	166.0 (1–549)
Increasing capacity of detecting rabid and contacted dogs	20.1 (1–103)	1–166	36.0% (33.0–39.1)	149.1 (1–578)
Increasing capacity of capturing stray dogs	22.5 (1–117)	1–175	39.2% (36.2–42.3)	162.3 (1–574)
Increasing capacity of emergency vaccination	22.7 (1–114)	1–160	39.2% (36.2–42.3)	161.6 (1–534)

To assess the contribution of stray dogs in an epidemic further, the relationship between outbreak size and the proportion of rabid dogs that were stray was examined using a generalised linear model with quasi-binomial errors, using the Ibaraki model with vaccination coverage 0%, and 1000 iterations.

## Results

### Parameter estimation

Estimated parameter values for rabid dog movement (*α, δ, ε*) from fitting the model to the historical Osaka outbreak data are reported in Table 2. We found no significant difference between numbers of observed and simulated rabies cases (*P* = 0.8).

### Risk assessment

**Fig. 2.**
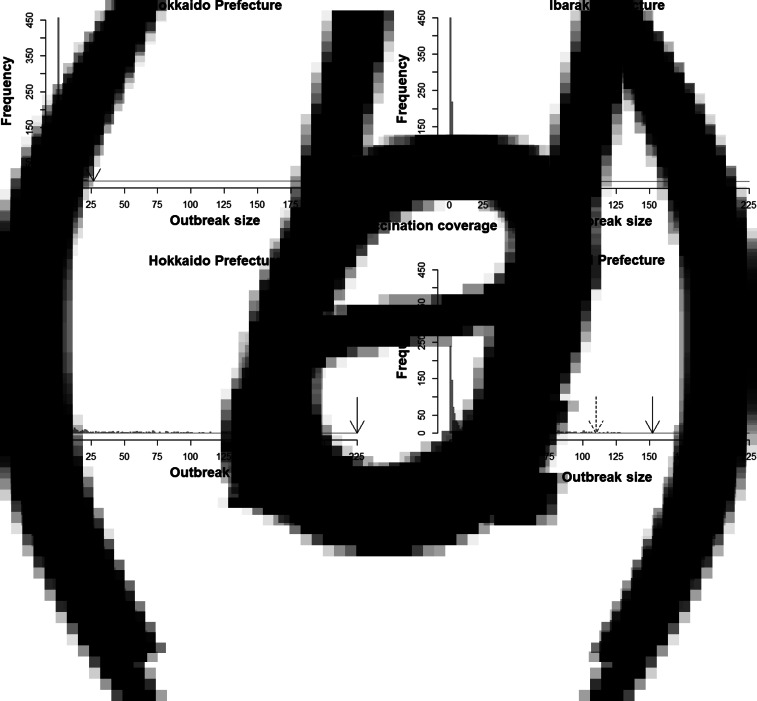
Predicted outbreak sizes in Hokkaido (a) and Ibaraki (b) Prefectures under current vaccination coverage, and without vaccination (c and d). Dashed and solid arrows shows the 97.5th percentile and maximum of final size.

The mean outbreak sizes, including the index cases, in Hokkaido and Ibaraki Prefectures under current vaccination coverage were 3.1 (median 2; range 1–27; 95% CI 1–14) and 4.7 (median 2; range 1–106; 95% CI 1–37), respectively (Fig. 2a and b), with 6.9% and 9.0% of outbreaks ⩾10 cases. The mean epidemic durations were 55.3 days (median 31; range 1–537; 95% CI 1–269) and 68.2 days (median 31; range 1–610; 95% CI 1–369) in respective prefectures.

In a scenario in which annual mandatory vaccination was discontinued, outbreaks were larger, with a mean of 22.8 (median 4; range 1–225; 95% CI 1–142) and 21.7 (median 4; range 1–152; 95% CI 1–110) rabid dogs in Hokkaido and Ibaraki Prefectures (Fig. 2c and d, Table 4), with 35.5% and 35.1% of outbreaks ⩾10 cases.

The increase of time between the onset of the index dog and the detection from 30 to 90 days increased the final outbreak size, but the probability of outbreaks ⩾10 cases remained unchanged (Table 4).

Emergency responses (increasing capacities for detecting rabid and rabies-contacted dogs, capturing stray dogs and performing emergency vaccinations) did not affect the outbreak sizes, or the proportion of outbreaks with ⩾10 cases (Table 4).

In the 116 records of bite injuries caused by individual rabid dogs, the average number of people bitten by a rabid dog was 5.5 (median 3; range 1–46; 95% CI 1–29). The mean numbers of rabid dog bite injuries per incursion in Hokkaido and Ibaraki Prefectures under current vaccination coverage were estimated to be 4.4 (median 0; range 0–210; 95% CI 0–35) and 6.7 (median 0; range 0–312; 95% CI 0–58), respectively, by the stochastic approach, and 2.6 (median 2; range 1–22; 95% CI 1–12) and 3.9 (median 2; range 1–88; 95% CI 1–31), respectively, by the ratio approach.

### Sensitivity analysis

**Fig. 3.**
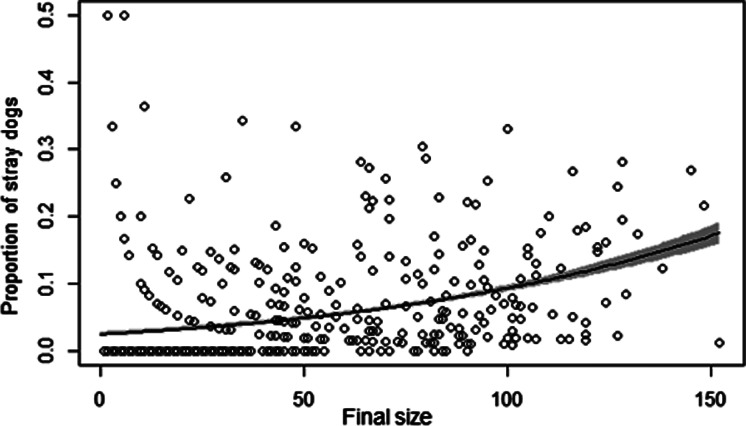
Relationship between the final outbreak size and the proportion of stray dogs among dog rabies cases using the Ibaraki Prefecture model with preferential biting of stray dogs under 0% vaccination coverage. The solid line is the predicted value based on a logistic regression, and grey area shows the 95% confidence interval of the regression parameters.

Table 5 shows the sensitivity analysis results. The smaller *R*_0_ produced smaller epidemic sizes than the default, but even *R*_0_ 1.6 still showed the possibility of over 10 cases. Discontinuation of mandatory vaccination or further decrease of vaccination coverage to 30% was sensitive to the epidemic size, though the size was still limited. Epidemic sizes were sensitive to the probability of unintentionally releasing a rabid owned dog when the probability was increased to 90%. However, smaller probabilities did not change the epidemic sizes very much. Assuming rabid stray dogs were more likely to contact other stray dogs rather than owned dogs had little impact on outbreak sizes when the result of default 50% was compared with the scenario of the probability of selection of stray dogs to bite based on the proportion of stray dogs out of total dog population (0.9%). However, the scenario that a rabid stray dog selects stray dog to bite at 90% chance produced larger outbreak (mean 11.3 cases). In the separate analysis using GLM, a positive relationship was observed between outbreak size and the proportion of stray dogs among rabies cases in the vaccination coverage 0% setting (slope of logit = 0.01, standard error = 0.0008, *P* < 0.01, Fig. 3).

**Table 5.** Sensitivity analysis results

Parameter	Value space	Mean (95% CI)	Range
Reproduction number	1.2	1.9 (1–6)	1–15
	1.6	2.7 (1–11)	1–51
	2.0	3.9 (1–23)	1–89
	2.42^a^	4.7 (1–37)	1–106
Vaccination coverage	0%	21.7 (1–110)	1–152
	30%	11.4 (1–75)	1–124
	51.8%^a^	4.7 (1–37)	1–106
	70%	3.2 (1–17)	1–82
Probability of releasing a rabid owned dog	5%	3.1 (1–11)	1–62
	10%	3.3 (1–13)	1–114
	50%^a^	4.7 (1–37)	1–106
	90%	12.0 (1–81)	1–126
Probability that a rabid stray dog selects a stray dog to bite	0.9%	3.8 (1–18)	1–50
	10%	4.0 (1–19)	1–53
	50%^a^	4.7 (1–37)	1–106
	90%	11.3 (1–154)	1–204

a Default values for Ibaraki model with current vaccination coverage.

## Discussion

Risk assessment using mathematical modelling is useful for planning disease control options, as models can demonstrate the potential outcomes of an introduction of an infectious disease and the changes on risk achieved by application of counter measures. Our study provides important messages, regarding responses to, and contingency planning for rabies incursions.

The risk of an incursion resulting in an outbreak of 10 or more dog rabies cases was assessed to be between 6.9% and 9.0% under current vaccination coverage and dog-keeping practices in Japan. Outbreaks would therefore typically be limited, but due to the extended rabies incubation period (15.5 days) [21], once secondary cases occur, the duration of the epidemic can become long.

The final size of outbreaks was strongly affected by reactions of dog owners if their dogs developed rabies. Even under the current vaccination coverage, a 90% chance of releasing rabid dogs could prolong the epidemic to more than a year. Thus, sensitisation of dog owners about rabies and recommended actions when rabies is detected is critical. Direct reporting of suspicious signs of rabies to local authorities may be better than bringing symptomatic dogs to veterinary clinics, as such transport incurs additional risks. Veterinary clinicians should, of course, also be reminded to suspect the possibility of rabies.

Our results showed that mandatory vaccination for prevention of rabies limits the risk from incursions. *R*_0_ reported worldwide is generally smaller than the value we used [17], and sensitivity analysis on *R*_0_ showed that the outbreak size may be even smaller. Smaller epidemic size in Hokkaido Prefecture than in Ibaraki Prefecture under current vaccination coverage may be due to higher vaccination coverage in Hokkaido, as the epidemic size under vaccination coverage 0% in Hokkaido was larger than that in Ibaraki. However, the cost-effectiveness of mandatory vaccination should be carefully discussed, as it incurs expenses for both dog owners and public services, and the risk of rabies introduction is very low [8]. Obviously, accumulated cost of mandatory vaccination over many years including future is bigger than that of contingency measures, which had not incurred since elimination in Japan. On the other hand, costs of control can be large without mandatory vaccination as epidemic size may be larger at an incursion. Thus, discussions on rabies control options taking risks of both rabies introduction and spread into account are needed. Most rabies-free countries, such as UK [22, 23], France [24] and Australia [25], do not use mandatory dog vaccination.

Our assessment of the efficacy of rabies control measures did not show any clear benefit to strengthening contingency plans. By default, we modelled a delay of 30 days from detection to initial response. It is assumed that an even earlier initial response, i.e. immediate capture of the primary case would prevent secondary cases. The result showed that once initial response was delayed for a month, further delay would not markedly change the final outbreak size. It should be noted that in settings where rabies outbreaks have been reported in previously rabies-free areas, delays to detection and response have far exceeded 60 days [26], which was the worst-case scenario that we examined. Scenarios with increased capacities for finding contacted dogs, and implementing emergency vaccination employed realistic assumptions. In the case of a dog rabies outbreak, once it has spread to a dog population, it may become challenging to contain in a short period, unless response capacity is dramatically strengthened, as realistic strengthening of contingency plans did not substantially change the final size in the model. The relationship between the outbreak sizes and the proportion of strays among rabid dogs suggested that larger epidemics are likely to be associated with rabies spread among the stray dog population. The sensitivity analysis also showed that when rabid stray dogs spread the disease within the sub-population, outbreak size may become larger. Control of stray dogs is therefore an important defence against rabies spread. The effect of stray dog population control was shown during historical outbreaks in Osaka Prefecture [13].

As suggested in a study in Tanzania [20], not all rabid dogs bite people, and the estimated number of bite injury cases by rabid dogs would be small under the current situation. However, under a small probability of large-scale outbreak, large number of bite injury cases may occur, as numbers of dog bite injury cases were positively correlated with dog rabies cases in historical outbreaks in Osaka [13]. A previous study found that 0–60% of people develop rabies after being bitten by a rabid dog if untreated, and the probability depends on the site of bite [27]. Awareness of rabies is thought to be low among Japanese citizens; only half of Japanese travellers bitten by stray or domestic dogs in rabies-endemic countries obtained post-exposure prophylaxis [28]. Therefore, given these vulnerabilities associated with low awareness, introduction of a rabid dog to Japan would pose a non-trivial risk of human death.

There are four points to be discussed with regard to the validity of our mathematical model. First, we used *R*_*0*_ from the historical epidemic in Osaka Prefecture, where many stray and unleashed dogs were present. It is therefore likely that contact rates may have been higher than they are currently, however, evidence suggests that *R*_*0*_ does not depend on dog population density [29]. In current Japan, most dogs are kept inside, and the risk of rabies spread would be underestimated if *R*_*0*_ is assumed to be density-dependent. We assumed frequency-dependent transmission and therefore that a rabid dog in present day Japan would infect the same number of dogs as in the past. Instead of changing *R*_*0*_ to reflect the difference of dog population density from the past, we did model the probability of unintentionally releasing a rabid dog, because all owned dogs are leashed in present day Japan. We used the default 50% for this probability; however, owned dogs in Japan are generally paid good attention, and the probability may be lower. The sensitivity analysis showed relatively small change in the outbreak size at 5% and 10% scenarios for this probability.

The second point bearing on the validity of our model is our method for determining the geographical locations of cases. The fitted parameter *α* that relates the distance kernel and dog density had an influence of 50% on determining case locations. We are limited in our estimation, in that we did not have the exact case locations or knowledge of who-bit-whom from the historical data, but our results indicate that in the setting of a large-scale epidemic, more cases will be observed in areas with high dog population densities. It should be noted that this dog population dependency is on the geographical allocation of rabid dogs, and not about the value of *R*_0_, which was discussed above. The adjustment parameter (*ε* = 1.1) from the distance kernel showed that distances between index and secondary cases were comparable between Osaka and Tanzania [15].

The third point is about the probability of a rabid dog biting at least one person. This information is not available in Japan, and we used data from Tanzania [20]. The number of bite injuries simulated using these data was comparable to the results using the bite–dog rabies ratio in the past Osaka outbreak [13], suggesting reasonable prediction.

Another limitation is that our model does not take into account recreation activities that might facilitate rabies transmission in modern society, such as running facilities for dogs and dog socialisation in parks. Our results should therefore be interpreted with caution, as transmissions in potential hotspots could result in unexpectedly large epidemics.

In conclusion, following a rabies incursion into Japan, outbreak sizes are expected to be limited. Reactions of owner to their dog developing rabies were the most influential factor for outbreak control. Rabies education should therefore be targeted to veterinarians, veterinary students and pet owners, as well as to the general public. Our results suggest that outbreak response capacity is currently strong, but that maintenance efforts should not be relaxed. Discussions of mandatory vaccination maintenance should be encouraged, and such discourse must acknowledge and evaluate overall risks considering both introduction and spread of rabies, and cost-effectiveness.
